# Does Thermal Variability Experienced at the Egg Stage Influence Life History Traits across Life Cycle Stages in a Small Invertebrate?

**DOI:** 10.1371/journal.pone.0099500

**Published:** 2014-06-09

**Authors:** Kun Xing, Ary A. Hoffmann, Chun-Sen Ma

**Affiliations:** 1 Climate Change Biology Research Group, State Key Laboratory for Biology of Plant Diseases and Insect Pests, Institute of Plant Protection, Chinese Academy of Agricultural Sciences, Beijing, China; 2 Departments of Zoology and Genetics, Bio21 Institute, The University of Melbourne, Parkville, Victoria, Australia; Oxford Brookes University, United Kingdom

## Abstract

Although effects of thermal stability on eggs have often been considered in vertebrates, there is little data thermal stability in insect eggs even though these eggs are often exposed in nature to widely fluctuating ambient conditions. The modularity of development in invertebrates might lead to compensation across life cycle stages but this remains to be tested particularly within the context of realistic temperature fluctuations encountered in nature. We simulated natural temperate fluctuations on eggs of the worldwide cruciferous insect pest, the diamondback moth (DBM), *Plutella xylostella* (L.), while maintaining the same mean temperature (25°C±0°C, 25±4°C, 25±6°C, 25±8°C, 25±10°C, 25±12°C) and assessed egg development, survival and life history traits across developmental stages. Moderate fluctuations (25±4°C, 25±6°C) did not influence performance compared to the constant temperature treatment, and none of the treatments influenced egg survival. However the wide fluctuating temperatures (25±10°C, 25±12°C) slowed development time and led to an increase in pre-pupal mass, although these changes did not translate into any effects on longevity or fecundity at the adult stage. These findings indicate that environmental effects can extend across developmental stages despite the modularity of moth development but also highlight that there are few fitness consequences of the most variable thermal conditions likely to be experienced by *Plutella xylostella*.

## Introduction

Because natural diurnal fluctuations in temperature can impose different impacts on ectotherms when compared to constant temperatures [1], it is important to assess the impact of thermal stability on ectotherm performance [2]. In organisms with complex life histories, temperature fluctuations at one life cycle stage might affect phenotypes at other stages [3]. Fluctuations are likely to be particularly important at the egg stage because this stage is frequently exposed to diurnal fluctuating temperatures in nature [4] and cannot escape thermal extremes in the same way that larvae and adults can behaviorally thermoregulate.

A number of studies have explored the effects of egg temperatures on life history performances in vertebrate ectotherms [5] and these studies have shown that egg temperature impacts egg traits [6] and also post-hatching traits [7]. On the other hand, invertebrates are different from vertebrate ectotherms by exhibiting a modularity of life cycles that may assist in dealing with environmental variability by possible insulation of later stages from disturbances during embryogenesis [8]. The egg stage of invertebrates is thought to be susceptible to thermal stability because of rapid rates of heat transfer and short durations [9] that make them sensitive to thermal fluctuations involving brief high temperature exposure [10] and recovery normal temperature intervals [11].

Short term stress exposures have been used to explore the thermal stress effects on the egg stage [12], although these types of studies do not consider the effects of daily fluctuations that might lead to cumulative effects through injury when stressful low night temperatures are followed by stressful daytime temperatures [13], [14]. In some relevant studies, changes to temperature regimens involved both means and fluctuating ranges simultaneously [15], making it hard to separate out the effects of fluctuations *per se*. Moreover the extent to which fluctuating conditions at the egg stage influence later stages is largely unexplored.

The diamondback moth (DBM), *Plutella xylostella* (Lepidoptera: Plutellidae), is the most destructive pest of cruciferous vegetables, and is widely distributed from tropical to cool temperate regions where substantial fluctuations in temperature are experienced [16], [17]. In this species it is well known that changes in constant temperatures affect life history traits such as growth, development, survival, reproduction [18] and migration [19].

Here we consider the following questions: (1) Do thermal fluctuations of the magnitude experienced in nature during egg stage affect egg hatch in DBM? (2) Do any fluctuations experienced at the egg stage have subsequent effects at the larval, pupal or even adult stage? We incubated eggs of DBM at a constant temperature of 25°C and five fluctuating temperature of 25±4°C, 25±6°C, 25±8°C, 25±10°C and 25±12°C and considered effects on development time and survival of eggs, larval and pupal development time, larval growth rate and survival, adult longevity and fecundity.

## Materials and Methods

### Insect rearing

The population of DBM larvae was collected from Brassica fields in May 2010, with the permission of the Experiment Station of Hubei Academy of Agricultural Sciences, in Wuhan, Hubei Province, China. All stages of DBM were reared in an insect rearing room with artifical diets (Southland Products Incorporated, USA) at a constant 25°C, 50%–70% RH and a photoperiod of L16:D8. DBM had been reared under these conditions for at least 10 generations before this study. After pupation, 200 pupae (1∶1 sex ratio) [20] were transferred to a screen cage (35×20×35 cm) for adult emergence. Once adults emerged, a cotton ball immersed in 10% honey water solution was supplied for adult feeding. 93 female adults eclosed and laid eggs. Two pieces of laboratory film (7×5 cm) were immersed in fresh juice of cabbage leaves for 3–5 seconds and then hung from the top of the screen cage at 15:00–16:00 h for egg laying. The film laid with DBM eggs was taken out at 7:00–8:00 am the next day. About 300 eggs on the film were counted and put on the surface of artificial diets (120 g) in a plastic rearing box (10×10×9 cm). The rearing box was kept at the rearing room until pupation and then followed the same procedures that were described above for pupae and adults rearing.

### Design and manipulation of fluctuating temperature cycles

**Figure 1:**
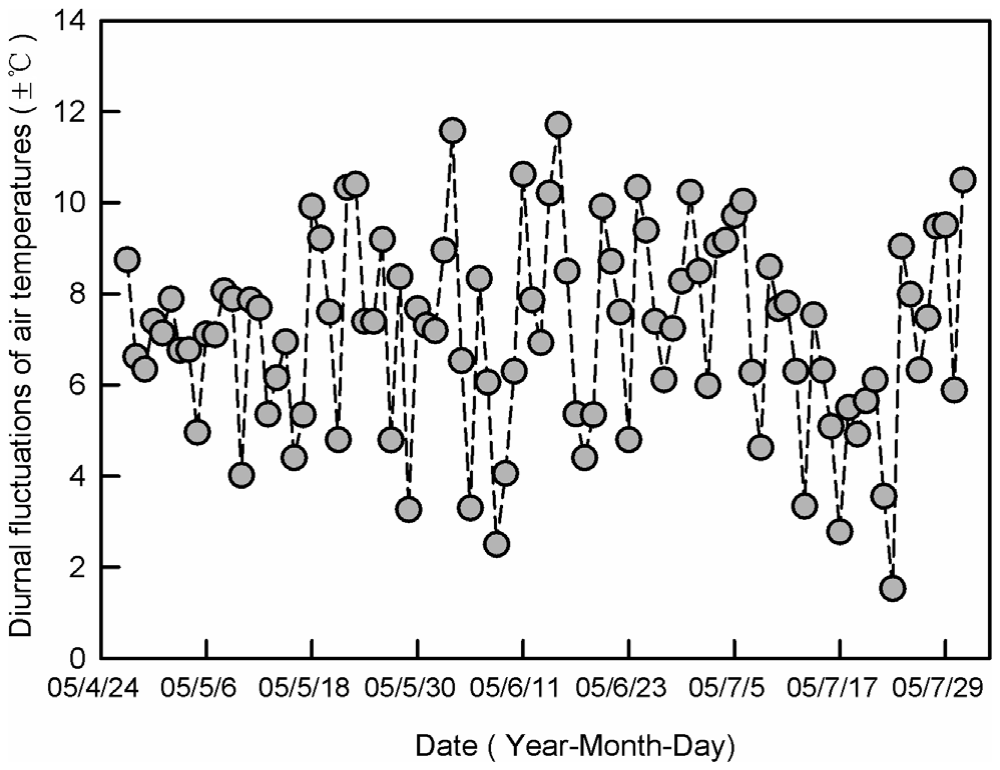
Diurnal fluctuation of air temperatures in a cabbage field in Beijing. Temperature was recorded with a data logger placed close to the underside of leaves and placed 30–35 cm above the ground in the middle of a 2 ha cabbage field.

A 24-h temperature cycle was used to simulate temperature fluctuations in a cabbage field in summer. Daytime temperature lasted for 8 hours (08:00–16:00) with the daily maximum temperature (DTmax) at 14:00, and the night temperature lasted for 16 hours (16:00–08:00) with the daily minimum temperature (DTmin) at 4:00. Temperature changed gradually between DTmax and DTmin. One constant temperature (25°C) and five fluctuating temperatures were used: 25±4°C, 25±6°C, 25±8°C, 25±10°C and 25±12°C. We selected 25±12°C as the widest diurnal range, because the temperatures in the microhabitat which DBM would experience in cabbage fields fluctuated daily within this range in summer in Beijing (Fig. 1). In addition, the mean temperature experienced in these fields is around 25°C.

**Figure 2:**
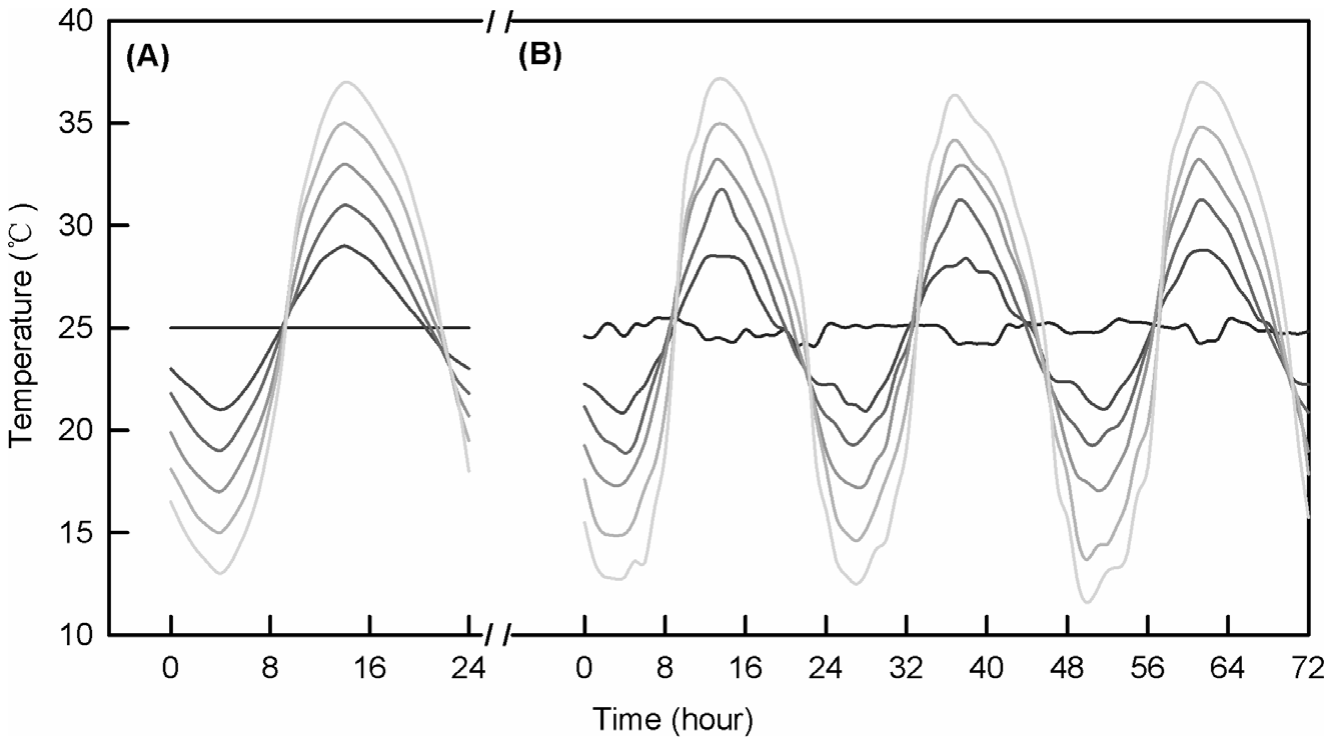
Target (A) and recorded temperatures (B) in experiments. Target (A) and recorded temperatures with different fluctuating ranges around 25°C in climate chambers for three consecutive days (B).

Temperatures in different climate chambers were logged every 20 minutes (U23-001, Hobo Ltd., USA) and showed that average temperatures in the chambers were around 25°C (Table1, Fig. 2 A–B). Relative humidity in chambers was 50%–70% and photoperiod was set to 16∶8 (L∶D) during the experiment.

**Table 1:** Target and actual recorded temperatures with different fluctuating ranges around 25°C in climate chambers.

Target Temperature(°C)	Recorded temperature	
	Mean (°C)	Fluctuating range(±°C)
25±0	24.86±0.19	0.55±0.09
25±4	24.95±0.21	3.77±0.14
25±6	24.91±0.20	6.22±0.37
25±8	25.17±0.17	8.01±0.13
25±10	24.80±0.18	10.18±0.33
25±12	25.05±0.17	12.10±0.25

Note: X±SD represent means and their standard errors.

### Growth, survival and reproduction

Plates were sterilized for 30 min by ultraviolet light, and each well was filled with 1.7 ml prepared artificial diet for DBM feeding. Three new eggs (laid within the last 12 h by 1–2 days old females) were transferred into each well with a fine camel's hair brush. To facilitate ventilation, the plates were covered with fine nylon mesh. Three plates were placed in each climate chamber. Over 200 (3 eggs*24 wells*3 plates = 216) eggs were observed in each temperature regimen. Hatching status of eggs was checked (with a stereo microscope) twice daily at 08:00 and 20:00, because temperatures in climate chambers at these times were similar to room temperature (about 25°C).

After eggs hatched, all tested insects were moved to a 25°C rearing room. Newly hatched larvae from different treatments were randomly assigned to wells (1 larva per well). Ninety six larvae (24 wells *3 plates  = 72) were used for each temperature treatment. The pupation status was checked daily at 08:00 until all larvae had died or developed to pupae. When larvae developed into pre-pupa [21], they were weighed. Plates were renewed every three days to keep artificial diets fresh. When larvae became spindle-shaped and had a thin white silk cocoon, they were recorded as having entered the pupa stage [22]. Pupae continued to be observed at 08:00 am until all adults emerged or pupae died (based on lack of adult emergence). Survival and development time were recorded for all immature stages. Newly emerged male and female adults which were from the same replicate under the same egg temperature treatment were paired and transferred into a glass tube (3×12 cm) for mating and egg-laying. Two sides of the tube were covered with the fine stainless steel mesh for ventilation. A piece of cotton immersed with honey water solution (10%) was placed in the tubes for adult feeding. At 15:00–16:00, a piece of laboratory film was inserted to the tube for egg laying. The adults were transferred into a new tube with new film at 7:00–8:00 every day until females died. All eggs laid on the film and inner surface of the tube were counted.

### Statistical analysis

Effects of different temperature treatments during egg stage on all indices of growth, survival and reproduction were analyzed with ANOVA in which egg temperatures and adult sex were treated as fixed factors, and replicate plates as a random factor (nested within egg temperatures). Means were compared using Duncan's multiple range test in SAS V8 (SAS Institute, Cary, NC). We analyzed effects of different temperature treatments during the egg stage on development time of eggs/larvae/pupae, growth rate of larvae, percent hatching rate (number of larvae/number of eggs×100), percent pupation rate (number of pupae/number of larvae×100), percent emergence rate (number of adults/number of pupae×100), adult fecundity and longevity. Growth rate of larvae were expressed as mg/d (pre-pupal mass/development duration of larvae).

## Results

### Egg development and survival

**Figure 3:**
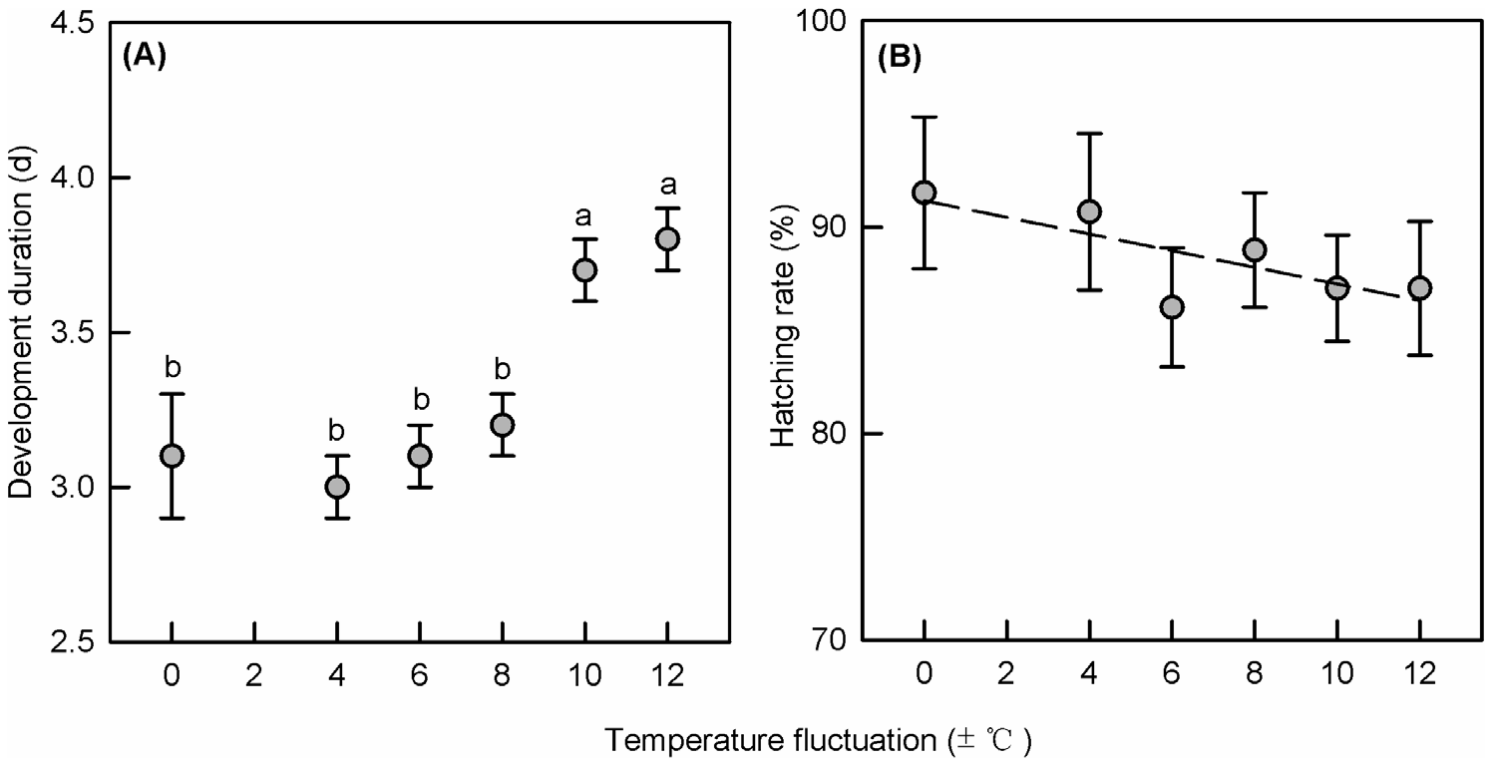
Egg traits (Mean±SE) after different treatments. (A) Development time of eggs and (B) hatching rate of DBM under six different temperature regimens with the same mean temperature but six different fluctuating ranges (25±0°C, 25±4°C, 25±6°C, 25±8°C, 25±10°Cand 25±12°C). Different letters at the top of columns indicate significant differences at P = 0.05.

Egg development was not affected by either sex (F_1,219_ = 0.041, P = 0.840) or replicate plates (F_2,219_ = 3.199, P = 0.061), but significantly by egg temperatures (F_5,219_ = 57.213, P<0.001) (Table 2). Eggs incubated at constant or moderately fluctuating temperatures (25±0°C, 25±4°C, 25±6°C and 25±8°C) developed significantly faster than those incubated at wider fluctuating temperatures (25±10°C and 25±12°C) (Fig. 3A). Overall, hatching success was higher than 83% and did not differ significantly between egg temperature treatments (F_5,18_ = 0.454, P = 0.802, Fig. 3B) and replicate plates (F_2,18_ = 0.558, P = 0.589) (Table 2). However, there was a trend for larger fluctuations to decrease hatchability (y  =  −0.403x +91.270, R^2^  =  0.505, P<0.0001).

**Table 2:** Results of ANOVA testing the effects of egg temperature treatments, sex, and replicate plates (as a random factor, nested within egg temperatures) on development time of egg, larva or pupa, pre-pupal mass, larval growth rates, adult longevity, female fecundity, hatching rate, pupation rate and emergence rate in *Plutella xylostella* (L.).

Trait	Source	df	MS	*F*	*P*
Egg development time	Egg treatment (ET)	5,219	25.478	57.213	**0.000**
	Replicate plates	2,219	1.425	3.199	0.061
	Sex (S)	1,219	0.018	0.041	0.840
	ET x S	5,219	0.077	0.174	0.972
Hatching rate	Egg treatment	5,18	15.121	0.454	0.802
	Replicate plates	2,18	18.574	0.558	0.589
Pre-pupal mass	Egg treatment (ET)	5,219	1000.872	16.876	**0.000**
	Replicate plates	2,219	332.623	5.608	**0.004**
	Sex (S)	1,219	36.863	0.622	0.531
	ET x S	5,219	72.183	1.217	0.302
Larval growth rate	Egg treatment (ET)	5,219	16.676	14.007	**0.000**
	Replicate plates	2,219	12.089	10.154	**0.000**
	Sex (S)	1,219	0.007	0.006	0.940
	ET x S	5,219	0.809	0.679	0.640
Larval development time	Egg treatment (ET)	5,219	0.336	0.687	0.634
	Replicate plates	2,219	1.327	2.710	0.069
	Sex (S)	1,219	0.576	1.177	0.279
	ET x S	5,219	0.465	0.950	0.450
Pupation rate	Egg treatment	5,18	26.642	0.226	0.943
	Replicate plates	2,18	6.652	0.061	0.941
Pupal development time	Egg treatment (ET)	5,219	0.198	0.333	0.893
	Replicate plates	2,219	0.067	0.112	0.894
	Sex (S)	1,219	0.106	0.179	0.673
	ET x S	5,219	0.418	0.703	0.622
Emergence rate	Egg treatment	5,18	34.324	0.332	0.882
	Replicate plates	2,18	183.762	1.777	0.219
Adults longevity	Egg treatment (ET)	5,219	6.760	0.925	0.466
	Replicate plates	2,219	78.348	10.722	**0.000**
	Sex (S)	1,219	278.663	38.134	**0.000**
	ET x S	5,219	3.690	0.505	0.772
Female fecundity	Egg treatment	5,96	177.242	0.226	0.950
	Replicate plates	2,96	1602.680	2.048	0.135

Significant P-values are given in bold.

### Larval growth, pe-pupal mass, development and survival

**Figure 4:**
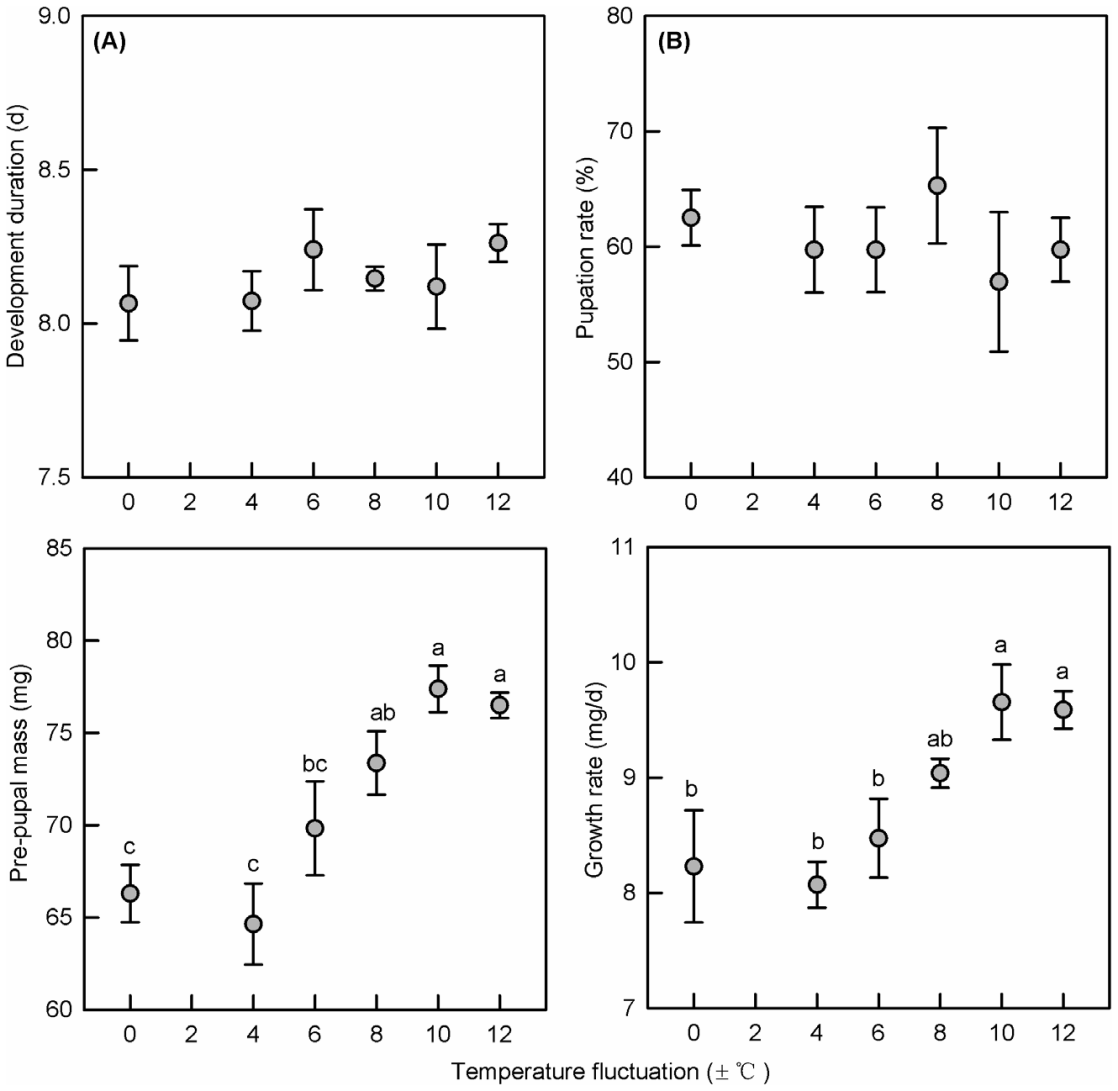
Larva traits (Mean±SE) after different treatments. (A) Development time of larva, (B) pupation rate, (C) pre-pupal mass and (D) larval growth rate following emergence from eggs exposed to different fluctuating temperature ranges (25±0°C, 25±4°C, 25±6°C, 25±8°C, 25±10°C and 25±12°C). Different letters at the top of columns indicate that significant differences exist between two treatments at P = 0.05.

The pre-pupal mass was affected significantly by egg temperature treatments (F_5,219_ = 16.876, P<0.001, Fig. 4C); there was also a significant impact of replicate plates (F_5,219_ = 5.608, P<0.01) but not sex (F_5,219_ = 0.622, P = 0.531) (Table 2). Larval growth followed a similar treatment pattern to pre-pupal mass (F_5,219_ = 14.007, P<0.001, Fig. 4D). Larvae that hatched from the eggs incubated at 25±10°C and 25±12°C grew significantly faster (ca 1.0 mg/d) than those from eggs incubated under smaller fluctuations. There was no difference in growth rate of larvae from eggs held at 25±10°C and 25±12°C, or from eggs held under lower fluctuations. Larval development was not significantly influenced by egg treatments (F_5,219_ = 0.687, P = 0.634, Fig. 4A), and there was no significant effects of replicate plates (F_2,219_ = 2.710, P = 0.069) or sex (F_1,219_ = 1.177, P = 0.279). Pupation rate was not affected by egg treatment (F_5,18_ = 0.226, P = 0.943, Fig. 4B) or replicate plates (F_2,18_ = 0.061, P = 0.941) (Table 2)

### Pupal and adult traits

**Figure 5:**
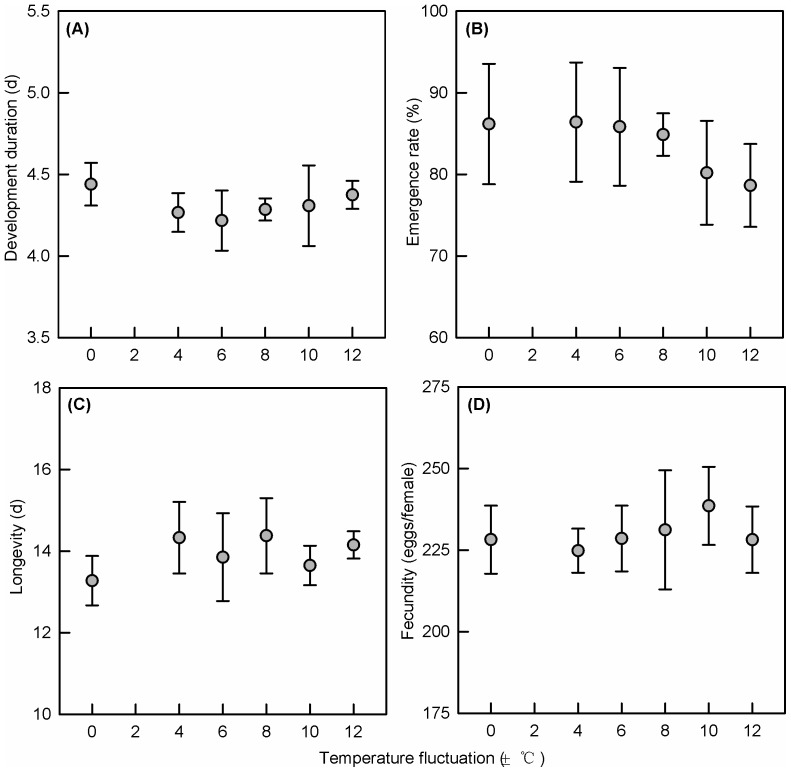
Pupa and adult traits (Mean±SE) after different treatments. (A) Development time of pupa, (B) emergence rate, (C) Adults longevity and (D) female fecundity in the egg of DBM following emergence from eggs exposed to different fluctuating temperature ranges (25±0°C, 25±4°C, 25±6°C, 25±8°C, 25±10°Cand 25±12°C).

No pupal or adult traits were influenced by the egg treatments (Table 2). There were no differences in pupal development time (F_5,219_ = 0.333, P = 0.893, Fig. 5A), pupal emergence rate (F_5,18_ = 0.332, P = 0.882, Fig. 5B), adult longevity (F_5,219_ = 0.925, P = 0.466, Fig. 5C) or fecundity (F_5,96_ = 0.226, P = 0.950, Fig. 5D). Replicate plates affected adult longevity (F_5,219_ = 10.722, P<0.001), while adult longevity differed significantly between the sexes (F_1,219_ = 38.134, P<0.001).

## Discussion

### 1. Effects of thermal fluctuations on eggs

Temperature fluctuations affected development time of the egg stage, but only at high fluctuations of ±10°C. This suggests that a temperature range of 17–33°C may not have much impact on egg development time if the mean temperature is 25°C. The impact of thermal stability on egg development is thought to be related to the upper and lower thermal limit for egg development [23]. Within an optimum range, developmental responses to temperature are expected to be approximately linear [24]. Daytime temperatures higher than the mean temperature and thus accelerate egg development, but the nighttime temperatures lower than the mean and slow development. With regular sinusoidal fluctuating temperatures as in our experiments, a uniform rate of development of DBM eggs is expected under moderate fluctuations in temperature, in contrast to fluctuations encompassing extreme values that depress development [25]. According to Jensen's inequality and the Sharpe-DeMichele model, development should slow if daytime temperature falls over the upper threshold or nighttime temperature falls below the lower threshold for development [26]. The maximum temperature of 37°C in our experiment is higher than 34°C, the upper threshold for development of DBM [27], for 6 hours (0.25 day) per day. Such high temperatures not only fail to compensate slower development at lower nighttime temperatures (<20°C), but may also depress development [27]. If we assume that there is no development above the upper threshold, we can largely account for the slower egg development at wide thermal fluctuations. Development time at 25±12°C, 3.86d is very close to the sum (3.79 d) of development time at 25±8°C, 3.04 d and no development duration (3 d x 0.25 d^−1^  =  0.75 d) at 25±12°C.

In contrast to effects on development time, temperature fluctuations had little impact on egg survival. Previous work has shown that egg survival is impacted by high temperature [28] and stress exposure time [29]. Hatching rates of DBM eggs are not changed much within a constant 8–31°C temperature range, but decreased to 58% at 32°C [30] or a slightly higher temperature [17]. However, in our results DBM eggs survived well even when temperature fluctuated as widely as ±12°C in which the daily maximum reached 37°C, far over the constant upper lethal 34°C limit. This high survival may relate to recovery from heat injury during optimum nighttime temperatures [31] or inadequate damage to affect survival. Similar patterns of recovery under fluctuating temperatures have been found in other species [32], [33]. Wide fluctuations of temperature may improve survival under heat stress but delay egg development [34]. Under a diurnal fluctuation cycle, daytime high temperatures induce the rapid synthesis of protective factors such as hsp70, mannitol and sorbitol, increasing heat resistance [35], [36], while nighttime temperatures reduce metabolism and energy consumption [37] and provide optimal thermal conditions for heat injury recovery.

### 2. Effects of egg treatments across developmental stages

Our results indicate that egg thermal stability significantly impacted the growth of larvae. Body mass and growth rate increased significantly in larvae hatched from eggs which experienced wide fluctuations. Compensatory growth can occur if organisms suffer from nutritional stress [38] or temperature stress [39] during early development. Wide temperature fluctuations in the egg stage may be stressful and trigger compensatory growth to increase larval development time. Stress during organisms' early stage does not change the number of cells [40], but results affect energy metabolism and utilization [41]. Stress in the egg stage often induces larval compensatory growth through accelerating food intake or increasing intake time (the latter extending development) [42]. Our results indicate development time was not extended (Fig. 4A, 5A), but body mass was increased (Fig. 4C) perhaps reflecting an increase in food intake. Compensatory growth during the larval stage may therefore be a life history strategy in DBM to buffer the impact of variable thermal conditions during the egg stage.

Thermal stability did not change other traits in larva, pupa or at the adult stage. The lack of long term fitness effects may reflect successful compensation and/or the fact that these insects undergo complete metamorphosis, such that different stages experience a physiological and morphological body rebuild during metamorphosis, creating a high degree of independence for each stage [8]. This may also help explain why early stage stress in complete metamorphosis insects does not affect adult fluctuating asymmetry [43], whereas such effects are detected in species without complete metamorphosis [44].

### 3. Pest management implications

Survival and phenology are important components of population models for monitoring, forecasting, control and harvesting in resource or pest management. Because the performance of organisms under [45] constant temperature regimens are well known in many species [46], [47], it is important to assess thermal conditions under which data from constant temperature experiments can be applied. Our results indicate that the traditional linear model for “constant temperature- development” can be widely applied if the mean temperature is optimal and the daily fluctuation is not very wide such as a mean of 25°C and fluctuation less than ±8°C for DBM [45]. However, these models may not describe development in an area or period with low or high mean temperature and/or wide diurnal temperature range. Survival data obtained at constant temperatures appear applicable to a wider range of temperatures. For practical applications, we suggest that when temperatures range between 17–33°C at the egg stage, the results from constant temperature experiments are applicable, but the diurnal temperature range should be incorporated into the development model and perhaps survival model when temperatures are known to fluctuate widely [48].

Recently, trends and future climate scenarios suggest a reduction in diurnal temperature ranges across the world [49]. This indicates that diurnal temperature range, as an independent factor, has potential impacts on population dynamics of DBM. In low latitude areas such as southern China [50] and southwestern Europe [51], the diurnal temperature range is below ±10°C at which the development of DBM can hardly be influenced and is similar to that at constant. However, in high latitude areas such as northwest China and central Russia (http://cdc.cma.gov.cn/home.do), the diurnal temperature range is above ±10°C at which the development of DBM may be restricted and is slower than at constant temperature. With diurnal temperature range decreasing in high latitude areas in the future, we presume that the development of DBM could be accelerated and consequently the number of generations could be increased. In this case, the potential risk of DBM will be increase during the growing season of *Brassica* crops.

## References

[1] Liu SS, Meng XD (1999) Modelling development time of *Myzus persicae* (Hemiptera: Aphididae) at constant and natural temperatures. Bull Entomol Res 89: 53–63. 10.1017/s0007485300000468 11020792

[2] Huey RB, Kingsolver JG (1989) Evolution of thermal sensitivity of ectotherm performance. Trends Ecol Evol 4: 131–135. 21227334 10.1016/0169-5347(89)90211-5

[3] Micheli-Campbell MA, Gordos MA, Campbell HA, Booth DT, Franklin CE (2012) The influence of daily temperature fluctuations during incubation upon the phenotype of a freshwater turtle. J Zool 288: 143–150.

[4] Booth DT (2006) Influence of incubation temperature on hatchling phenotype in reptiles. Physiol Biochem Zool 79: 274–281. 16555187 10.1086/499988

[5] Andrewartha SJ, Mitchell NJ, Frappell PB (2010) Does incubation temperature fluctuation influence hatchling phenotypes in reptiles? A test using parthenogenetic geckos. Physiol Biochem Zool 83: 597–607. 20477533 10.1086/652245

[6] Du WG, Shen JW, Wang L (2009) Embryonic development rate and hatchling phenotypes in the Chinese three-keeled pond turtle (*Chinemys reevesii*): The influence of fluctuating temperature versus constant temperature. J Therm Biol 34: 250–255.

[7] Metcalfe NB, Monaghan P (2001) Compensation for a bad start: grow now, pay later? Trends Ecol Evol 16: 254–260. 11301155 10.1016/s0169-5347(01)02124-3

[8] Potter KA, Davidowitz G, Arthur Woods H (2011) Cross-stage consequences of egg temperature in the insect *Manduca sexta* . Funct Ecol 25: 548–556.

[9] Angilletta MJ (2009) Thermal adaptation: a theoretical and empirical synthesis: Oxford University Press. 35–87.

[10] Huey RB, Bennett AF (1990) Physiological adjustments to fluctuating thermal environments: an ecological and evolutionary perspective. In: Morimoto RI, Tissieres A, Georgopoulos C, editors. Stress proteins in biology and medicine. New York, USA. Cold Spring Harbor Laboratory Press. 37–59.

[11] Berger D, Walters R, Gotthard K (2008) What limits insect fecundity? Body size-and temperature-dependent egg maturation and oviposition in a butterfly. Funct Ecol 22: 523–529.

[12] Roux O, Le Lann C, van Alphen JJM, van Baaren (2010) How does heat shock affect the life history traits of adults and progeny of the aphid parasitoid *Aphidius avenae* (Hymenoptera: Aphidiidae)? Bull Entomol Res 100: 543–549. 20102660 10.1017/S0007485309990575

[13] Pétavy G, David JR, Debat V, Gibert P, Moreteau B (2004) Specific effects of cycling stressful temperatures upon phenotypic and genetic variability of size traits in *Drosophila melanogaster* . Evol Ecol Res 6: 873–890.

[14] Rohmer C, David JR, Moreteau B, Joly D (2004) Heat induced male sterility in *Drosophila melanogaster*: adaptive genetic variations among geographic populations and role of the Y chromosome. J Exp Biol 207: 2735–2743. 15235002 10.1242/jeb.01087

[15] Terblanche JS, Nyamukondiwa C, Kleynhans E (2010) Thermal variability alters climatic stress resistance and plastic responses in a globally invasive pest, the Mediterranean fruit fly (*Ceratitis capitata*). Entomol Exp Appl 137: 304–315.

[16] Furlong MJ, Wright DJ, Dosdall LM (2013) Diamondback moth ecology and management: problems, progress, and prospects. Annu Rev Entomol 58: 517–541. 23020617 10.1146/annurev-ento-120811-153605

[17] Shirai Y (2000) Temperature tolerance of the diamondback moth, *Plutella xylostella* (Lepidoptera: Yponomeutidae) in tropical and temperate regions of Asia. Bull Entomol Res 90: 357–364. 11020794 10.1017/s0007485300000481

[18] Umeya K, Yamada H (1973) Threshold temperature and thermal constants for development of the diamond-back moth, *Plutella xylostella* L., with reference to their local differences. Jpn J Appl Entomol Zool: 112–123.

[19] Xing K, Ma CS, Han JC (2013) Evidence of long distance migration of diamondback moth (DBM) *Plutella xylostella*: a review. Chinese J Appl Ecol 24: 1769–1776. 24066569

[20] Chen YX, Tian HJ, Wei H, Zhan ZX, Huang YQ (2011) A simple method for identifying sex of *Plutella xylostella* (Linnaeus) larva, pupa and adult. Fujian J Agri Sci 26: 611–614.

[21] Sarfraz RM, Dosdall LM, Keddie AB, Myers JH (2011) Larval survival, host plant preferences and developmental responses of the diamondback moth *Plutella xylostella* (Lepidoptera: Plutellidae) on wild brassicaceous species. Entomol Sci 14: 20–30.

[22] Golizadeh A, Kamali K, Fathipour Y, Abbasipour H (2009) Effect of temperature on life table parameters of *Plutella xylostella* (Lepidoptera: Plutellidae) on two brassicaceous host plants. J Asia Pac Entomol 12: 207–212.

[23] Andrews RM, Schwarzkopf L (2012) Thermal performance of squamate embryos with respect to climate, adult life history, and phylogeny. Biol J Linn Soc 106: 851–864.

[24] Escobar A, Gil R, Ricardo Bojacá C, Jiménez J (2012) Modeling egg development of the pest *Clavipalpus ursinus* (Coleoptera: Melolonthidae) using a temperature-dependent approach. Insect Sci 19: 657–665.

[25] Estay SA, Clavijo-Baquet S, Lima M, Bozinovic F (2011) Beyond average: an experimental test of temperature variability on the population dynamics of *Tribolium confusum* . Popul Ecol 53: 53–58.

[26] Ruel JJ, Ayres MP (1999) Jensen's inequality predicts effects of environmental variation. Trends Ecol Evol 14: 361–366. 10441312 10.1016/s0169-5347(99)01664-x

[27] Golizadeh A, Kamali K, Fathipour Y, Abbasipour H (2007) Temperature-dependent development of diamondback moth, *Plutella xylostella* (Lepidoptera: Plutellidae) on two brassicaceous host plants. Insect Sci 14: 309–316.

[28] Qayyum A, Zalucki MP (1987) Effects of high temperature on survival of eggs of *Heliothis armigera* (Hübner) and H. *punctigera Wallengren* (Lepidoptera: Noctuidae). Aust J Entomol 26: 295–298.

[29] Ma CS, Hau B, Poehling HM (2004) Effects of pattern and timing of high temperature exposure on reproduction of the rose grain aphid, *Metopolophium dirhodum* . Entomol Exp Appl 110: 65–71.

[30] Chen FZ, Liu SS (2003) Development rate of *Plutella xylostella* L.(Lepidoptera: Plutellidae) under constant and variable temperatures. Acta Ecol Sin 23: 688–694.

[31] Zhao F, Zhang W, Hoffmann AA, Ma CS (2014) Night warming on hot days produces novel impacts on development, survival and reproduction in a small arthropod. J Anim Ecol doi: 10.1111/1365–2656.12196 10.1111/1365-2656.12196 24372332

[32] Yang Y, Stamp NE (1995) Simultaneous effects of night-time temperature and an allelochemical on performance of an insect herbivore. Oecologia 104: 225–233. 28307359 10.1007/BF00328587

[33] Davis JA, Radcliffe EB, Ragsdale DW (2006) Effects of high and fluctuating temperatures on *Myzus persicae* (Hemiptera: Aphididae). Environ Entomol 35: 1461–1468.

[34] Gharib B, de Reggi M, Connat JL, Chaix JC (1983) Ecdysteroid and juvenile hormone changes in *Bombyx mori* eggs, related to the initiation of diapause. FEBS lett 160: 119–123.

[35] Salvucci ME (2000) Sorbitol accumulation in whiteflies: evidence for a role in protecting proteins during heat stress. J Therm Biol 25: 353–361. 10838174 10.1016/s0306-4565(99)00107-2

[36] Zerebecki RA, Sorte CJB (2011) Temperature tolerance and stress proteins as mechanisms of invasive species success. PLoS ONE 6: e14806. 21541309 10.1371/journal.pone.0014806 PMC3082523

[37] Boardman L, Sørensen JG, Terblanche JS (2013) Physiological responses to fluctuating thermal and hydration regimes in the chill susceptible insect, *Thaumatotibia leucotreta* . J Insect Physiol 59: 781–794. 23684741 10.1016/j.jinsphys.2013.05.005

[38] Yengkokpam S, Sahu NP, Pal AK, Debnath D, Kumar S, et al (2013) Compensatory growth, feed intake and body composition of *Labeo rohita* fingerlings following feed deprivation. Aquac Nutr 19: 297–303.

[39] Macqueen DJ, Robb DHF, Olsen T, Melstveit L, Paxton CGM, et al (2008) Temperature until the ‘eyed stage’of embryogenesis programmes the growth trajectory and muscle phenotype of adult Atlantic salmon. Biol Lett 4: 294–298. 18348956 10.1098/rsbl.2007.0620 PMC2610038

[40] Pitts GC (1986) Cellular aspects of growth and catch-up growth in the rat: a reevaluation. Growth 50: 419. 3297935

[41] Metcalfe NB, Monaghan P (2003) Growth versus lifespan: perspectives from evolutionary ecology. Exp Gerontol 38: 935–940. 12954479 10.1016/s0531-5565(03)00159-1

[42] Ozanne SE, Hales CN (2002) Early programming of glucose–insulin metabolism. Trends Endocrinol Metab 13: 368–373. 12367817 10.1016/s1043-2760(02)00666-5

[43] Stoks R, Córdoba-Aguilar A (2012) Evolutionary ecology of *Odonata*: a complex life cycle perspective. Annu Rev Entomol 57: 249–265. 21910638 10.1146/annurev-ento-120710-100557

[44] Dmitriew C, Rowe L (2005) Resource limitation, predation risk and compensatory growth in a damselfly. Oecologia 142: 150–154. 15372227 10.1007/s00442-004-1712-2

[45] Worner SP (1992) Performance of phenological models under variable temperature regimes: consequences of the Kaufmann or rate summation effect. Environ Entomol 21: 689–699.

[46] Stevens MM (1998) Development and survival of *Chironomus tepperi* Skuse (Diptera: Chironomidae) at a range of constant temperatures. Aquat Insects 20: 181–188.

[47] Hassan MW, Dou W, Chen L, Jiang HB, Wang JJ (2011) Development, survival, and reproduction of the psocid *Liposcelis yunnaniensis* (Psocoptera: Liposcelididae) at constant temperatures. J Econ Entomol 104: 1436–1444. 21882714 10.1603/ec11046

[48] Paaijmans KP, Blanford S, Bell AS, Blanford JI, Read AF, et al (2010) Influence of climate on malaria transmission depends on daily temperature variation. Proc Natl Acad Sci 107: 15135–15139. 20696913 10.1073/pnas.1006422107 PMC2930540

[49] Braganza K, Karoly DJ, Arblaster JM (2004) Diurnal temperature range as an index of global climate change during the twentieth century. Geophys Res Lett 31: 219–231.

[50] Chen TX, Chen X (2007) Variation of diurnal temperature range in China in the past 50 Years. Plateau Meteor 1: 17–26.

[51] Makowski K, Wild M, Ohmura A (2008) Diurnal temperature range over Europe between 1950 and 2005. Atmos Chem Phys 8: 6483–6498.

